# Cockroaches as Vectors of Pathogens and Antimicrobial Resistance: Evidence from Healthcare, Community, and Agricultural Settings

**DOI:** 10.3390/insects17030310

**Published:** 2026-03-13

**Authors:** Assia Derguini, Nosiba S. Basher

**Affiliations:** 1 Microbial Ecology Laboratory, Faculté des Sciences de la Nature et de la Vie, Abderrahmane MIRA University, Bejaïa 06000, Algeria; assia.derguini@univ-bejaia.dz; 2 Biology Department, College of Science, Imam Mohammad Ibn Saud Islamic University (IMSIU), Riyadh 11623, Saudi Arabia

**Keywords:** antimicrobial resistance, cockroaches, hospital-acquired infections, insecticide resistance, multidrug-resistant bacteria, vector

## Abstract

Synanthropic cockroaches, especially *Blattella germanica* and *Periplaneta americana*, are persistent pests of human dwellings, healthcare facilities, food establishments, farms, and transport infrastructure. Accumulating field and laboratory studies indicate that synanthropic cockroaches carry clinically important bacteria, fungi, and parasites, including multidrug-resistant strains harbouring extended-spectrum β-lactamase, carbapenemase, and other antimicrobial-resistant determinants. Cockroaches acquire these organisms from sewage, waste, food residues, animal excreta, and contaminated clinical environments, and retain them on the cuticle and within a complex gut microbiota. Dissemination is predominantly mechanical, via contact transfer and deposition of regurgitate and faeces on food, equipment, and surfaces, but may be amplified by gut colonisation, microbial interactions, and horizontal gene transfer within the cockroach microbiome. In hospitals, cockroaches can connect high-burden reservoirs (drains, waste areas, kitchens) with vulnerable units, including intensive care units (ICUs), neonatal intensive care units (NICUs), burn units, and haemato-oncology wards. In food and livestock systems, they may contaminate housing, ingredients, and finished products, enabling spillover along supply chains and at ports. This review synthesises current evidence and highlights the following priorities: integrate cockroaches into infection prevention, food safety, and biosecurity; incorporate cockroach sampling into antimicrobial resistance (AMR) and genomic surveillance; and advance mechanistic research on cockroach–microbiota–pathogen interactions to improve pest management and safely explore cockroach-derived antimicrobial compounds. In this review, we distinguish external mechanical carriage (cuticular contamination) from internal gut carriage; we use “gut colonisation” only when persistence/replication or prolonged shedding is demonstrated.

## Simple Summary

Cockroaches, particularly the German cockroach and American cockroach, are common pests found in hospitals, homes, food facilities, and farms. These insects can carry dangerous bacteria, fungi, and parasites on their bodies and in their digestive systems, including drug-resistant “superbugs” that do not respond to common antibiotics. Cockroaches pick up these harmful microorganisms from sewage, garbage, contaminated food, and hospital environments, then spread them to clean surfaces, food, and medical equipment through contact, their droppings, and regurgitated material. In hospitals, cockroaches can move germs from dirty areas like drains and waste rooms to vulnerable patient units such as intensive care wards and burn units, potentially causing serious infections. In food production and livestock facilities, they can contaminate food products and spread disease along supply chains. This review examines current scientific evidence showing how cockroaches spread pathogens and antimicrobial resistance and calls for stronger pest control measures in healthcare, food safety programmes, and disease surveillance systems. The review also discusses the need for more research on how microorganisms interact with cockroaches and how to safely study potential antimicrobial compounds that cockroaches produce, which might one day help develop new antibiotics.

## 1. Introduction

Hospital-associated infections involving drug-resistant pathogens are substantial, with increased incidence of carbapenem-resistant *Acinetobacter baumannii*, methicillin-resistant *Staphylococcus aureus* (MRSA), and third-generation cephalosporin (3GC)-resistant *Enterobacterales (Cre)* [1]. These infections lead to longer hospital stays, increased need for intensive care, and higher mortality, straining healthcare resources, especially in regions with limited infection control measures [2,3]. The rise in antimicrobial resistance (AMR), coupled with the spread of infections through environmental reservoirs, has intensified focus on the role of insects, rodents, and companion animals as vectors of multidrug-resistant (MDR) bacteria. Insects, such as houseflies and cockroaches, can spread AMR pathogens across hospital environments, emphasising the need for better infection control measures [4,5]. Recent entomological and microbiological syntheses increasingly characterise synanthropic cockroaches as important vectors for clinically significant pathogens [6,7,8].

Historically, cockroaches were often conceptualised in medical and veterinary entomology as primarily “nuisance pests”, associated with disgust, allergens, and minor food spoilage rather than as substantive vectors of infectious disease. This view contrasted with the clear recognition of mosquitoes, triatomines, sand flies, and tsetse as “major” vectors of parasitic or viral pathogens, and may have contributed to the limited formal integration of cockroach control into infection–prevention policies. More recent syntheses, however, collating literature from hospitals, households, and food processing sites, have highlighted the breadth of pathogen taxa associated with cockroaches [9]. In a scoping review by Luckyjane Molewa et al., the authors concluded that cockroaches frequently harbour clinically important bacteria, including multidrug-resistant strains, on both the external cuticle and in the gut, and that these insects have been repeatedly implicated as potential mechanical contributors to hospital and household contamination [10]. The distinction between mechanical and biological transmission is central to understanding the epidemiological significance of cockroaches. Mechanical transmission refers to the passive transfer of pathogens acquired from contaminated substrates to new surfaces or hosts without replication or development of the pathogen within the vector, typically via contaminated mouthparts, cuticle, faeces, or regurgitated material. In contrast, biological transmission implies that the pathogen undergoes multiplication, development, or an essential part of its life cycle within the vector before being transmitted to a new host. While most evidence to date supports a predominantly mechanical role for cockroaches in spreading enteric and nosocomial bacteria, the demonstration of persistent gut colonisation and prolonged faecal shedding of certain pathogens suggests potential intermediate scenarios in which colonised cockroaches constitute living environmental reservoirs [5,9]. Risk assessment studies of ‘hygiene pests’ in hospitals indicate that cockroaches and filth flies can substantially amplify mechanical dissemination of bacteria in high-risk units, despite lacking classical biological vector competence [5].

Terminology used in this review: “external mechanical carriage” refers to microorganisms detected on the cockroach cuticle (e.g., surface washes/swabs of legs, tarsi, mouthparts, or whole-body rinses), whereas “gut carriage” refers to microorganisms recovered from dissected digestive tracts or gut contents. Detection in gut samples from cross-sectional field surveys indicates internal carriage but does not by itself confirm stable gut colonisation; we therefore reserve “colonisation” for studies that demonstrate persistence/replication and/or prolonged faecal shedding (e.g., longitudinal sampling or experimental infection models) [11].

Given the growing recognition of cockroaches as carriers of clinically important pathogens and MDR bacteria in diverse environments, there is a need for an updated synthesis that integrates entomological, microbiological, and epidemiological perspectives. Existing reviews have either focused broadly on bacteria associated with cockroaches, including potential biotechnological applications [9], or have scoped the literature on cockroach-borne bacteria with antibiotic resistance without systematically linking these findings to hospital epidemiology, food safety, and One Health frameworks [10]. Parallel work on insects, rodents, and pets as AMR reservoirs underscores the importance of integrating pest ecology into AMR surveillance and risk assessment, but provides limited cockroach-specific granularity [9]. The present review, therefore, aims to build on and extend this evidence base by focusing specifically on synanthropic cockroaches as vectors, reservoirs of pathogens, and antimicrobial resistance.

## 2. Taxonomy and Major Species

Modern systematics places cockroaches within the order Blattodea, which now encompasses both “traditional” cockroaches and termites following robust molecular and phylogenomic evidence that Isoptera is nested within Cryptocercidae + other cockroaches [12,13]. Within Blattodea, the superfamily Blattoidea includes Blattidae, Lamproblattidae, Tryonicidae, Anaplectidae, and Cryptocercidae + Isoptera, while the family Ectobiidae (formerly Blattellidae) contains many of the smaller household pests such as *Blattella germanica* and *Supella longipalpa* [13]. Synanthropic cockroaches, species that complete all or most of their life cycle in human-made structures, represent only a small fraction of total diversity: of approximately 4500 described cockroach species worldwide, about 30 are closely associated with human activity, yet these few species dominate medical and veterinary concern [8]. Phylogenomic studies have clarified relationships among medically important cockroaches and documented repeated transitions into indoor habitats, implicating a limited group of Ectobiidae and Blattidae as the principal contributors to public health risk [8,12].

Within this synanthropic assemblage, *B. germanica* (German cockroach; Ectobiidae) is the quintessential domiciliary and hospital pest, characterised by small body size (≈10–15 mm), rapid development, and strict dependence on warm indoor environments [8]. Population-genomic data from 281 *B. germanica* sampled across 17 countries suggest a hybrid origin from *Blattela asahinai* in southern Asia ~2100 years ago and subsequent global dissemination along trade and transport networks [14]. In contrast, *Periplaneta americana* (American cockroach; Blattidae) is among the largest synanthropic species (adult length typically 30–40 mm) and exhibits a more peridomestic distribution, thriving in sewer systems, service ducts, boiler rooms, and other warm, humid voids that connect sanitation infrastructure with human-occupied spaces [8]. A Khorramabad-based entomological survey found that *P. americana* comprised about two-thirds of captured cockroaches across hospitals, households, and restaurants, indicating its role in linking distinct indoor environments [15]. Other synanthropic cockroaches contribute substantially to indoor infestation patterns and may modulate pathogen and AMR risks across vertical and horizontal gradients within buildings. *Blatta orientalis* (oriental cockroach; Blattidae) typically favours cooler, damp basements, drainage channels, and waste storage areas; recent metabarcoding of cuticle and gut samples from urban populations in Santiago, Chile, confirmed its status as a globally distributed pest and revealed diverse bacterial communities, including both mutualistic symbionts and human pathogens [16]. In the same Khorramabad survey of Davari et al., *B. orientalis* comprised nearly 15% of captured cockroaches and, like co-occurring *B. germanica* and *P. americana*, exhibited high rates of bacterial contamination, illustrating how multiple Blattidae and Ectobiidae species can jointly seed environmental reservoirs of opportunistic pathogens in hospitals and restaurants [15]. *S. longipalpa* (brown-banded cockroach; Ectobiidae) is more often associated with drier, warmer upper stories and ceiling spaces, completing its entire life cycle indoors, with these taxa together representing over 80% of reads and likely contributing to adaptation to indoor microclimates [17].

## 3. Biology and Ecology of Medically Important Cockroaches: Development, Behaviour, and Habitat Use

Medically important synanthropic cockroaches are hemimetabolous insects, undergoing three main developmental stages (egg, nymph, and adult) without a pupal stage, with eggs packaged in chitinous oothecae that confer partial protection against desiccation and some insecticidal exposures [18]. Detailed laboratory work on *B. germanica* has reconstructed a canonical life cycle of roughly 95 days at 26 °C, with oothecae typically containing 30–40 embryos that develop over approximately 35 days before hatching into first-instar nymphs, which in turn pass through 5–6 nymphal instars before adult emergence after about 50 additional days [18,19].

In contrast, *P. americana* generally exhibits a slower developmental tempo and lower intrinsic rate of increase but compensates through larger body size, prolonged adult longevity, and exploitation of structurally complex sewer and service environments [20]. Experimental bioassays conducted in Antalya, Türkiye, in which field-collected *B. germanica* and *P. americana* were reared for entomopathogenic nematode susceptibility testing, report that *B. germanica* can complete its life cycle in as little as 2–3 months under favourable temperature and nutritional conditions, whereas *P. americana* typically requires 6–12 months to complete a generation, with females producing approximately 6–14 oothecae containing 14–16 embryos each over their lifespan [20]. The same study, designed primarily as a biological control evaluation, highlights that adult *P. americana* can persist for many additional months in laboratory and field settings, combined with a preference for cooler, humid microhabitats, enabling chronic colonisation of building infrastructure and increasing opportunities for acquisition and redistribution of environmental pathogens from drains, sewage and waste storage areas into human-occupied spaces [20].

Aggregation pheromones and faecal cues drive dense clustering of *B. germanica* and *P. americana* in cryptic harbourages such as cracks behind wall-mounted cabinets, voids under sinks, motor housings of refrigerators, and cable or pipe chases, where warmth, high humidity, and accumulated organic residues create favourable conditions for both cockroaches and microorganisms [21]. Meta-analytic synthesis of indoor trapping studies indicates that cockroach densities peak in kitchens and nearby service rooms, where food and moisture are concentrated [22]. Surveillance data from Chinese urban settings indicate that cockroach density indices are greatest in catering establishments and farmers’ markets and markedly lower in ordinary residential buildings, consistent with preferential occupation of warm, moist, nutrient-rich environments [23].

## 4. Cockroach Microbiota and Interaction with Pathogens

Synanthropic cockroaches harbour a dual microbial system consisting of the obligate intracellular endosymbiont *Blattabacterium* and a highly diverse hindgut microbiota acquired horizontally from the environment and conspecific faeces [18]. Metagenomic profiling of *B. germanica* hindguts has revealed a highly diverse community (25 phyla, >600 genera) dominated by Bacteroidota, Firmicutes, and Proteobacteria, with *Blattabacterium* restricted to bacteriocytes [24]. Longitudinal work in the same model demonstrates that hindgut communities assemble rapidly after hatching via coprophagy and environmental exposure, forming a relatively stable “core” consortium despite variation in diet and antibiotic history [18,25]. Comparable patterns are seen in *P. americana*: 16S rRNA surveys of laboratory and field populations show a stable core microbiome dominated by Firmicutes and Bacteroidota that is resilient to short-term changes in macronutrient composition, indicating that nutritional generalism in this species is underpinned by a robust gut consortium [26]. More recent work tracking the gut microbiota across life stages in *P. americana* found oothecae dominated by *Blattabacterium*, whereas nymphs and adults were enriched in taxa associated with carbohydrate and protein fermentation, reinforcing the view that symbiotic nutritional provisioning is conserved across medically important cockroaches [27]. Similar dual systems have now been described in other domiciliary species such as *S. longipalpa*, where amplicon sequencing of individual cockroaches revealed co-occurring *Blattabacterium* and a hindgut community dominated by Proteobacteria, Firmicutes, and Bacteroidota, suggesting a shared symbiotic architecture among urban pest cockroaches [17]. Collectively, these studies support a model in which *Blattabacterium* provides vertically transmitted, host-integrated nitrogen recycling, while the hindgut microbiota supplies a flexible, environmentally acquired interface with diets, contaminants, and pathogens encountered in human-dominated environments [18].

The functional importance of this dual symbiosis has been dissected experimentally by selectively perturbing either the endosymbiont or the hindgut microbiota. Rifampicin-based quasi-aposymbiotic lines of *B. germanica* showed a reduction in *Blattabacterium* load of up to five orders of magnitude; although the gut microbiota recovered compositionally after treatment, affected lines displayed severe fitness costs, including reduced fecundity and failure to sustain a third generation, indicating that the hindgut community cannot fully compensate for the loss of endosymbiont-mediated nitrogen recycling [28]. In a complementary design using kanamycin pulses, metagenomic profiling of *B. germanica* hindguts demonstrated that antibiotic treatment markedly reduced alpha diversity and disrupted co-occurrence networks, yet predicted functions involved in carbohydrate and protein metabolism, vitamin biosynthesis, and nitrogen transformations were largely maintained, implying substantial functional redundancy within the core community [25]. Repeated kanamycin perturbation experiments involving 512 *B. germanica* demonstrated recurrent reorganisation of gut communities with preservation of key metabolic functions and no measurable effects on host survival or reproduction [29]. When *Blattabacterium* was strongly depleted but the gut microbiota allowed to re-establish through coprophagy, adults in subsequent generations exhibited near-normal hindgut community structure but persistent deficits in nitrogen storage and growth, underscoring the distinct and non-interchangeable roles of endosymbiont and gut microbiota [30]. Together, these perturbation experiments demonstrate that the German cockroach’s microbiota is simultaneously plastic and constrained: compositional turnover is substantial under antibiotic pressure, but host-relevant metabolic functions and community-level redundancy are strongly conserved [18,24].

Beyond nutrition, gut microbes make measurable contributions to cockroach physiology and ecological performance in variable urban environments. In *P. americana*, experimental suppression of the gut microbiota through antibiotic treatment significantly reduced standard metabolic rate in adult females relative to untreated controls, suggesting that commensal microbes contribute to energy balance and the processing of complex dietary substrates common in human dwellings [31]. Life-stage-resolved surveys of *P. americana* showed that nymphs harbour microbiota enriched in fermentative and short-chain fatty acid-producing taxa, whereas adults exhibit increased representation of taxa associated with detoxification and nutrient scavenging, patterns that may facilitate developmental transitions in diet and habitat use in sewers, kitchens, and hospital basements [27]. For *S. longipalpa*, comparison of laboratory and field colonies revealed broadly similar community composition but significantly higher relative abundance of putative opportunistic pathogens and environmental bacteria in field-collected individuals, implying that urban cockroaches dynamically integrate local microbial assemblages into a conserved core template [17]. From an ecological perspective, such microbial buffering likely contributes to cockroaches’ ability to exploit nutrient-poor or toxic substrates in hospitals, food premises, and waste systems while maintaining population growth under intense anthropogenic stressors [18,31].

The cockroach gut microbiota is also tightly coupled to innate immunity and colonisation resistance against enteric pathogens. Genomic and transcriptomic analyses of *B. germanica* have revealed an expanded repertoire of pattern recognition receptors and antimicrobial peptide (AMP) genes, including multiple attacins, defensins, and the cockroach-specific blattellicins, compared with holometabolous insect vectors [19]. Detailed annotation of the immune deficiency (IMD) pathway and AMP expression patterns across tissues and haemolymph indicates that these effectors are poised to respond rapidly to bacterial challenge in the gut, fat body, and haemolymph, supporting a central role in regulating both symbionts and transient pathogens [32]. Within this biologically active hindgut reservoir, metagenomic studies reveal a dense “resistome” composed of antimicrobial resistance genes (ARGs) and mobile genetic elements distributed across commensal and opportunistic taxa. In the mSystems interkingdom analysis of *B. germanica* hindguts, Domínguez-Santos and colleagues catalogued a diverse ARG repertoire conferring predicted resistance to β-lactams, tetracyclines, aminoglycosides, glycopeptides, polymyxins, and folate synthesis inhibitors, with many linked to integrons, transposases, and plasmid-associated sequences even in cockroaches never exposed to clinical antibiotics, leading the authors to conclude that German cockroaches constitute natural reservoirs and potential vectors of ARGs [24]. In *Pycnoscelus surinamensis*, controlled experiments have demonstrated the social transmission of tetracycline-resistant genes between groups through contaminated faeces and substrate [33].

## 5. Cockroaches as Reservoirs of Antimicrobial Resistance

Observational and microbiological studies indicate that synanthropic cockroaches, primarily *B. germanica* and *P. americana*, establish long-term populations in human-built environments where they are continually exposed to human, animal, and environmental microbiota, as well as residual antimicrobials [33,34,35]. AMR denotes the ability of microorganisms to survive concentrations of antimicrobial agents that would normally inhibit or kill them, while MDR strains are commonly defined as those non-susceptible to at least one agent in three or more antimicrobial classes [36]. Rather than acting solely as transient mechanical carriers, cockroaches support persistent gut and cuticular communities that include resistant bacteria [4,10,37].

Culture-based surveys and genomic investigations have consistently demonstrated high carriage of MDR *Enterobacterales*, non-fermenting Gram-negative bacilli, and resistant Gram-positive cocci in and on cockroaches, with MDR proportions commonly exceeding half of all bacterial isolates and, in some instances, approaching universal resistance within specific taxa [38,39,40,41].

Importantly, the reported prevalence of MDR/ESBL/carbapenemase phenotypes varies not only with true local epidemiology but also with study design and laboratory methods. Differences in trapping strategy, sample processing (external wash vs. gut dissection; individual vs. pooled insects), culture/enrichment and screening approaches, antimicrobial panels, breakpoint standards (CLSI vs. EUCAST), and MDR definitions can shift estimates; therefore, comparisons across studies and across Table 1 and Table 2 should be interpreted cautiously.

### 5.1. Prevalence of MDR and Resistant Bacteria in Hospital Settings

Hospital surveys consistently report high bacterial carriage among trapped cockroaches, with many recovered isolates showing multidrug-resistant patterns such as ESBL and carbapenemase production [7,42]. In Southern Ethiopia, for example, cockroaches collected from multiple wards frequently yielded MDR Enterobacterales, including ESBL- and metallo-β-lactamase-producing *Klebsiella pneumoniae*, *Escherichia coli*, and *A. baumannii* (Table 1) [41]. Hospital surveys also identify MRSA carriage in cockroaches (e.g., Iranian hospitals; see Table 1 and Section 6.1). [43].

**Table 1:** Representative (non-exhaustive) list of antimicrobial-resistant and multidrug-resistant bacteria recovered from cockroaches in hospital settings.

Country	Cockroach Species (If Reported)	Sample Size	Bacterial Taxa Recovered	Prevalence of Bacterial Carriage (%)	Prevalence of MDR/ESBL/Carbapenemase Isolates (%)	Ref.
Southern Ethiopia	*B. germanica*; *P. americana*	245	*K. pneumoniae*; *E. coli*; *Enterobacter cloacae*; *P. aeruginosa*; *A. baumannii*; other Gram-negative bacilli	>90% of samples yielded Gram-negative bacteria	34.7% ESBL (42/121 isolates); 23.1% MβL (28/121); >90% MDR among Gram-negative isolates	[41]
Algeria	*B. germanica*	10	ESBL- and carbapenemase-producing Enterobacterales (*E. coli*, *K. pneumoniae*, *E. cloacae*)	NR (high carriage reported)	*bla_OXA-48_*; *bla_CTX-M-15_*	[44]
Algeria	*B. germanica*	10	*Pseudomonas putida*	10% (1/10 cockroaches yielded the focal isolate)	*bla_VIM-2_*	[45]
Iran	*B. germanica*; *P. americana*	530	*S. aureus* (external and gut isolates)	NR (*S. aureus* frequently recovered)	MRSA: 55.4% (external isolates) and 40.5% (gut isolates); most MRSA MDR	[43]
Iraq	*Periplaneta* spp.)	300	*K. pneumoniae*; *Proteus mirabilis*; *Enterobacter aerogenes*; *E. coli*; *A. baumannii*; *Serratia marcescens*; *Citrobacter freundii*; *S. aureus*	96.6%	NR	[35]
Bangladesh	*P. americana*	NR	Multiple antibiotic-resistant bacteria (NR; genomes reported)	NR	NR	[46]
Iran	*B. germanica*	109 cockroaches	*E. coli*	28.44%	*bla_NDM 4/31_*, *bla_OXA-48_*	[42]
Iran	*B. germanica*), *P. americana*), *B. orientalis*	660	*Streptococcus* spp.	13.48%	NR	[47]
Pakistan (Peshawar)	NR	527	MDRE/Enterobacterales (incl. *E. coli*, *K. pneumoniae*, *E. cloacae*, *K. aerogenes*, *C. freundii*, *E. hormaechei*)	88.8%	*bla_CTX-M-15_*, *bla_NDM_*, *bla_OXA-48-like_*	[48]
Morocco	NR	75	MDR Gram-negative bacteria and/or Enterobacterales (NR)	The prevalence of ESBL-producing and carbapenemase-producing GNB was 6.7 and 1.8%, respectively	*bla_CTX-M-28_*, *bla_NDM-1_*, *qnrS1*	[49]

NR, not reported or not explicitly quantified.

### 5.2. Prevalence of MDR and Resistant Bacteria in Community, Agricultural, and Border/Port Settings

Evidence from community settings shows that cockroaches collected from households, schools, and food premises frequently harbour MDR bacteria [10]. In Gondar, Ethiopia, domestic cockroaches yielded multiple MDR enteric bacteria, including *Salmonella*, *Enterobacter*, and *Shigella* spp. (Table 2) [38]. These findings, demonstrating substantial MDR prevalence in domestic cockroach-associated Gram-negative bacteria, align with broader syntheses identifying household insects, rodents, and companion animals as reservoirs of AMR determinants for both first-line and last-resort antimicrobials [4,10]. In Accra, Ghana, CRe were recovered from cockroaches in 15% of households, including ESBL-, AmpC- and carbapenemase-producing *E. coli*, *Klebsiella* and *Enterobacter* spp. (Table 2) [39].

A Tunisian survey spanning domestic and food service environments provides complementary evidence that cockroaches from non-hospital settings can harbour clinically significant plasmid-mediated resistance determinants. Cockroaches from these sites yielded ESBL-producing *Enterobacteriaceae*, including colistin-resistant *E. coli* (Table 2) [40]. In Kampala, Uganda, cockroaches collected from secondary school latrines and kitchens carried MDR enteric bacteria, including ESBL/AmpC-producing and carbapenem-resistant *E. coli* and enterococci (Table 2) [50].

**Table 2:** Examples of antimicrobial-resistant and multidrug-resistant bacteria recovered from cockroaches in community, agricultural, and border/port settings (non-exhaustive).

Country	Setting	Cockroach Species	Sample Size	Bacterial Taxa Recovered	Prevalence of Bacterial Carriage (%)	Prevalence of MDR/ESBL/Carbapenemase Isolates (%)	Key Resistance Features	Ref.
Ethiopia	Households in an urban community	NR (domestic cockroaches)	60	*Salmonella* spp.; *Enterobacter* spp.; *Shigella* spp.; *E. coli*; other Gram-negative bacilli	181 isolates from 60 cockroaches (multiple isolates per cockroach)	Overall 64.1% MDR; *Salmonella* 100% MDR; *Enterobacter* 90.5% MDR; *Shigella* 76.9% MDR	MDR mainly to ampicillin, 3GCs, tetracycline, cotrimoxazole and chloramphenicol	[38]
Ghana	Urban households: cockroach and human stool sampling	NR	100 households	3GC-resistant *E. coli*; *K. pneumoniae*; *Citrobacter freundii*; *Enterobacter agglomerans*; *Salmonella* Choleraesuis	CRe was detected in cockroaches in 15% of households	ESBL among cockroach CRe in 5% of households; most ESBL-positive isolates are MDR; 2 carbapenemase-positive isolates are XDR	*bla_CTX-M-15_*, *bla_TEM-24_*, *bla_SHV-3_*, *bla_NDM-1_*, *bla_OXA-48_*	[39]
Tunisia	Houses, collective catering facilities, and craft industries	NR	115 cockroaches	*E. coli*; *K. pneumoniae*; other Enterobacterales	144 Enterobacterales isolates from 115 cockroaches	15.3% ESBL (22/144); some colistin-resistant; all mcr-1-positive isolates MDR	*bla_CTX-M-1_*; *bla_CTX-M-15_*; colistin resistance via *mcr-1*	[40]
Uganda (Kampala)	Secondary schools; latrines, kitchens, and adjacent areas	Predominantly *P. americana*	168	*E. coli*; enterococci; *Salmonella* spp.; other enteric bacteria	NR	>30% MDR overall; ESBL/AmpC subset shows >90% MDR; carbapenem-resistant *E. coli* detected	MDR to β-lactams, fluoroquinolones, aminoglycosides, and sulfonamides; critical-priority carbapenem-resistant E. coli	[50]
Indonesia	Community/residential homes	*P. americana*	100	*E. coli*; *Klebsiella* spp.; *P. aeruginosa*; *Acinetobacter* spp.	NR	ESBL carriage: 14%	*bla_CTX-M_*	[51]
Iran	City-wide sampling (houses, restaurants and hospital environment)	*P. americana*; *B. germanica*	150	*Bacillus* spp.; *E. coli*; *P. aeruginosa*; *P. mirabilis*; *Klebsiella* spp.; *Citrobacter* spp.; CoNS; *Enterococcus* spp.; *S. aureus*	97.33%	NR	Highest resistance reported to cephalothin; intermediate resistance to ceftriaxone, ampicillin, amoxicillin-clavulanate, nalidixic acid and tetracycline	[15]

NR, not reported or not explicitly quantified.

### 5.3. Genotypic AMR Determinants in Cockroach-Associated Isolates

Available genomic and PCR-based studies of bacteria isolated from cockroaches have started to reveal the resistance genes and plasmid contexts associated with MDR and extensively drug-resistant (XDR) profiles [7]. In Tunisia, Landolsi et al. screened 144 *Enterobacteriaceae* isolates from 115 cockroaches and identified 22 ESBL producers, with most *bla_CTX-M-1_*-positive *E. coli* simultaneously harbouring the plasmid-borne colistin resistance gene *mcr-1* [40]. Multilocus sequence typing revealed seven *E. coli* sequence types, including ST648, a globally disseminated extraintestinal pathogenic lineage often linked to human and animal infections, indicating that cockroach-associated ESBL/*mcr-1 E. coli* belong to high-risk clones circulating across host species [40].

The Ghanaian household study provides some of the most detailed genotypic data on cockroach-borne cephalosporin-resistant *Enterobacterales*. Among 20 cockroach CRe isolates, these CRe carried typical CTX-M/TEM/SHV ESBLs and NDM/OXA-48 carbapenemases (see Table 2) [39]. Conjugation experiments showed efficient transfer of ESBL and carbapenemase genes to *E. coli* J53 recipients, often accompanied by co-transfer of resistance to cotrimoxazole, tetracycline, and ciprofloxacin [39]. In several households, paired cockroach and human isolates shared identical sequence types, ESBL alleles, and antibiograms, supporting the notion that cockroaches participate in local circulation of plasmid-mediated β-lactam resistance determinants within domestic microbiomes [39].

Hospital-based studies add further evidence that cockroach-associated bacteria may carry clinically important carbapenemase and ESBL determinants commonly encountered in high-acuity care. In Batna University Hospital, *B. germanica* captured from wards yielded CTX-M-15 and OXA-48-producing *Enterobacteriaceae*, indicating that German cockroaches can host both ESBL and class D carbapenemase genes known to drive hospital outbreaks [44]. From the same hospital’s burn unit, a non-fermenting *P. putida* isolate recovered from *B. germanica* carried *bla_VIM-2_* within a class 1 integron alongside the aminoglycoside-modifying enzyme gene *aac(6′)-Ib*, further highlighting the accumulation of carbapenemase and aminoglycoside resistance elements in cockroach-associated non-fermenters [45].

Viewed alongside broader animal and insect AMR syntheses, these molecular data suggest that cockroach-associated bacteria fit the general pattern observed in other animal reservoirs, where *bla_CTX-M_*, *bla_TEM_*, and *bla_SHV_* ESBL genes are frequently plasmid-encoded and co-located with resistance determinants for aminoglycosides, trimethoprim + sulfamethoxazole, and fluoroquinolones [4,36]. Coprophagy, aggregation behaviour, and movement across human, animal, and waste interfaces create repeated opportunities for horizontal gene transfer between bacterial populations inhabiting cockroach guts, cuticles, and faeces, although the directionality and frequency of gene exchange remain incompletely quantified [37,39,40]. Overall, post-2016 genomic studies, predominantly from North and West Africa and involving relatively small isolate numbers, nonetheless converge in showing that cockroach-associated microbiota can harbour high-risk ESBL, carbapenemase, and *mcr* genes in plasmid contexts similar to those circulating in human and livestock populations [39,40,41,44].

## 6. Cockroaches as Carriers of Human Pathogens in Hospital Environments

### 6.1. Bacterial Pathogens

A global systematic review and meta-analysis concluded that hospital-associated cockroaches harbour a diverse array of clinically important bacteria, with MDR isolates especially common on external surfaces [52]. Subsequent scoping and systematic reviews of pathogenic and antibiotic-resistant bacteria in cockroaches from healthcare facilities have confirmed repeated recovery of *Enterobacteriaceae*, *Pseudomonas aeruginosa*, *A. baumannii*, *S. aureus*, and enterococci in hospitals worldwide, frequently with multidrug-resistant or extensively drug-resistant phenotypes that parallel local clinical antibiograms [7,10].

A multi-setting study from Iraq that sampled 300 cockroaches from hospital wards, restaurants, and homes reported that 96.6% of individuals harboured bacteria; *E. coli* and other *Enterobacteriaceae* were common, and resistance was highest to cefotaxime, ampicillin, cephalothin, and kanamycin [35]. Together with earlier synthesis work highlighting that *B. germanica* and *P. americana* are among the most frequently contaminated species in hospitals, these surveys delineate a consistent pattern of Gram-negative colonisation in inpatient environments, with particularly dense cockroach infestations and diverse Gram-negative agents documented in paediatric, neonatal, and obstetric units [53,54].

Regional case studies from Africa and the Middle East illustrate the convergence between cockroach- and patient-associated AMR patterns. In southern Ethiopia, ESBL and MBL prevalence among cockroach-derived nosocomial pathogens was comparable to ESBL and carbapenemase frequencies in Enterobacterales causing surgical site infections in the same region, suggesting overlapping environmental and clinical resistome [41,55].

Cross-sectional surveys that process both external and internal compartments provide more granular insight into this gradient between mechanical and quasi-biological transmission. In an Iranian hospital study, 179 cockroaches (117 *P. americana*, 62 *B. germanica*) were trapped from wards and service areas; bacterial culture of both surface washes and dissected digestive tracts showed contamination in 173 insects (99.4%), with *E. coli* being the most frequently isolated species (26.6% of colonies), indicating that nearly all individuals functioned as multi-compartment bacterial carriers [56].

Using matrix-assisted laser desorption/ionisation time-of-flight mass spectrometry (MALDI-TOF MS), Mehainaoui and colleagues analysed both digestive tracts and external surfaces of 25 *B. germanica* collected in an Algerian healthcare facility, isolating 181 bacterial strains and achieving species-level identification for 96.5% of isolates (175/181) [57]. The majority of isolates were Gram-negative Enterobacteriaceae (103 strains across six genera, including *Citrobacter*, *Klebsiella*, *Kluvera*, *Leclercia*, *Morganella*, and *Serratia*), followed by *Pseudomonas* spp. (29.8%, 54 strains), with smaller proportions of *Staphylococcus* spp. (6.6%) and *Enterococcus* spp. (1.7%), and *Serratia marcescens* (44.2%) and *P. aeruginosa* (29.3%) were dominant; nearly half of all strains were recovered from cockroaches captured in maternity wards, with an additional quarter from paediatric wards, directly implicating high-risk patient care areas [57]. Abdolmaleki et al. collected 530 *P. americana* and *B. germanica* from hospitals and found that MRSA prevalence reached 52.8% in *P. americana* and 43.3% in *B. germanica*, with external washes of *P. americana* showing the highest MRSA contamination (59.6%) [43]. MRSA isolates from both external surfaces and gut contents displayed very high resistance to penicillin, ceftaroline, tetracycline (all 100%) and substantial resistance to gentamicin and trimethoprim–sulfamethoxazole, and carried multiple resistance genes including *blaZ*, *aacA-D*, *tetK*, *msrA*, *dfrA*, *ermA*, *gyrA*, *grlA*, and *rpoB* [43]. In a complementary 660-cockroach survey across Iranian hospitals, Chehelgerdi and Ranjbar reported that *Streptococcus pneumoniae*, *Streptococcus pyogenes*, and *Streptococcus agalactiae* were recovered from 6.96%, 4.82%, and 1.66% of specimens, respectively, and that streptococcal isolates harboured high frequencies of virulence genes such as *cfb*, *cyl*, *scaA*, and *glnA* alongside resistance determinants *pbp2b*, *pbp2x*, *mefA*, *ermB*, and *tetM* [47]. Phenotypically, these streptococci showed high resistance to tetracycline (80.9%), trimethoprim (65.2%), and penicillin (56.2%), illustrating that cockroaches can act as reservoirs not only of Gram-negative MDR pathogens but also of Gram-positive bacteria equipped with combinations of virulence and resistance traits relevant to severe invasive disease [47,53].

### 6.2. Fungi and Yeasts

Beyond bacteria, survey and culture-based studies have demonstrated that hospital-associated cockroaches frequently harbour opportunistic yeasts and moulds of clinical relevance [58]. A global meta-analysis by Nasirian found that approximately 84% of cockroaches from human dwellings were contaminated with fungi, encompassing 38 species from 19 families across three major pest taxa [52]. In a more recent systematic review focused on hospital cockroaches, fungi accounted for a substantial subset of pathogens reported, most frequently *Candida* spp. and *Aspergillus* spp. [7,21].

Hospital-based surveys indicate that a high proportion of trapped cockroaches carry culturable fungi, often with multiple species per insect [58]. In an Algerian tertiary hospital, Merad et al. trapped 100 cockroaches from six wards and cultured their external surfaces on Sabouraud dextrose agar; 78% of the insects carried fungi of medical importance, yielding 96 isolates dominated by *Rhizopus* spp. (21.9%), non-*albicans Candida* (16.7%), *Aspergillus niger* (15.6%), and *Lichtheimia* spp. (12.5%) [59]. Fungal contamination was significantly associated with specific hospital locations, including nephrology–haemodialysis, kitchens, toilets, and patient rooms [59]. A cross-sectional study from a national referral hospital in Tanzania captured hospital cockroaches from intensive care, burn, surgical, and paediatric oncology units as well as staff and central kitchens, and showed that external washes consistently yielded medically important yeasts and moulds, including *Candida albicans*, *Candida glabrata*, *Aspergillus fumigatus* and *A. niger*, indicating that even routine ward and kitchen infestations can sustain a rich mycobiota on cockroach cuticles [58]. Complementing these single-site surveys, an Iranian cross-sectional study comparing clinical and non-clinical environments in Khorramabad found that hospitals accounted for two-thirds of the trapped cockroaches and that 49.0% of hospital-derived specimens carried fungi on their external surfaces, mainly *Candida, Geotrichum*, and *Penicillium* spp., with *B. germanica* showing the highest proportion of fungal contamination (60.7%) [60].

In a hospital sewer system study from Iran, Khodabandeh et al. collected 55 *P. americana* from manholes connected to a teaching hospital and found that all specimens were infected with at least one fungal species; internal cultures yielded multiple opportunistic yeasts, notably *Candida krusei*, *Candida kudriavzevii*, *C. glabrata*, and other non-*albicans Candida*, alongside *A. niger*, *Penicillium*, *Mucor*, and *Rhizopus* spp. [61]. A separate cross-sectional survey of *P. americana* from residential dwellings in southwestern Iran demonstrated carriage of pathogenic fungi within the gut, including *C. albicans*, *C. tropicalis*, *A. fumigatus*, *A. niger*, and *Mucor* spp., suggesting that peridomestic cockroach populations can maintain viable fungal propagules in the digestive tract and potentially deposit them via faeces onto food-contact surfaces and floors [62]. The meta-analysis by Nasirian further quantified that internal contamination is generally more frequent and considered more hazardous than external contamination, reflecting the higher likelihood that ingested spores survive passage through the alimentary canal and are repeatedly shed into the environment [52]. Although relatively few studies have assessed antifungal susceptibility in cockroach-associated fungi, available data indicate that antifungal-resistant isolates are emerging, warranting increasing concern [63]. In the Tanzanian hospital study, *Candida* isolates recovered from cockroach washes were tested for fluconazole susceptibility using CLSI microdilution; a subset of non-intrinsically resistant species, including *C. albicans*, *C. glabrata*, and *Candida parapsilosis*, exhibited elevated minimum inhibitory concentrations consistent with fluconazole resistance, leading the authors to conclude that hospital cockroaches may act as environmental reservoirs for azole-resistant yeasts [58]. These observations align with broader clinical surveillance data showing a rise in fluconazole resistance among non-*albicans Candida* spp.; for example, a multicentre Chinese study of 519 *Candida tropicalis* isolates documented fluconazole resistance in 16.5% of strains, most of which were also cross-resistant to voriconazole [64]. Similarly, a global review has highlighted clonal outbreaks of fluconazole-resistant *C. parapsilosis* in hospitals, driven by ERG11 mutations and facilitated by the environmental persistence of the organism on surfaces and devices [65]. The overlap between the *Candida* species dominating candidemia and those recovered from hospital cockroaches raises a plausible One Health–type concern that insects may contribute to the local environmental pool of azole-resistant yeasts, even though direct transmission to patients has not yet been formally demonstrated.

### 6.3. Viruses and Parasites

Survey data based on microscopy and PCR show that cockroaches can carry a range of enteric viruses and intestinal parasites on their cuticle and within the gut [8]. A Chinese urban survey of *B. germanica* used multiplex RT-PCR on pooled gut and surface samples and reported a panel of human enteric viruses in cockroach pools, adding to earlier evidence that these insects can acquire viral particles from sewage-contaminated drains, refuse, and kitchen environments [21]. Narrative reviews on insect vectors in the context of COVID-19 concluded that previously reported detections of hepatitis viruses, enteroviruses and rotaviruses in cockroaches most likely represent environmental contamination rather than active infection [8,66]. Taken together, current survey and review data indicate that cockroaches can acquire and transport enteric viruses, with risk magnitude modulated by infestation density, access to faecal material and proximity to food-handling surfaces [8]. The COVID-19 pandemic has prompted more targeted work on SARS-CoV-2 in cockroaches, yielding apparently divergent but complementary findings on risk. In Shiraz, Iran, Kalantari et al. conducted a real-time RT-qPCR-based cross-sectional study of *B. germanica* collected from three hospitals and two dormitories, documenting SARS-CoV-2 RNA in a subset of field-caught cockroaches and arguing that contamination most likely reflected contact with virus-laden faeces or fomites in COVID-19 care areas [67]. In contrast, a U.S. household study placed 133 sticky and liquid-baited traps in 40 homes with confirmed human COVID-19 cases (including seven homes with PCR-positive pets), capturing 1345 insects from 11 Diptera families and Blattodea and testing 243 pools by RT-qPCR; none were positive for SARS-CoV-2 RNA [68]. Beyond bacteria, a parasitological survey in Cameroon found that domestic cockroaches carried a wide range of protozoan and helminth parasites, including *Entamoeba histolytica*, *Entamoeba dispar*, *Giardia duodenalis*, and geohelminth eggs, with a large proportion of insects harbouring at least one medically important parasite and thus providing additional routes for enteric infection if their bodies, faeces, or secretions contaminate food preparation areas [69].

Intestinal parasites of public health and veterinary importance have likewise been documented in cockroaches from community, market, and agricultural settings [70]. In Lagos, Nigeria, Adenusi et al. examined domiciliary cockroaches from human dwellings and identified a broad spectrum of human parasites on external surfaces and in guts, including *Blastocystis* spp., *Giardia* spp., *Entamoeba* spp., and various helminth eggs, highlighting risks in crowded households where cockroach activity overlaps with food preparation and children’s play areas [71]. A microscopy-based study in Thai fresh markets collected cockroaches from vegetable and meat stalls and reported high contamination with soil-transmitted helminths (e.g., *Ascaris* spp., *Trichuris* spp., hookworm) and protozoan cysts on both cuticular surfaces and gut contents, emphasising the potential for these insects to disseminate parasites along informal food chains [72]. From an explicitly veterinary perspective, an experimental-plus-field study in Thailand demonstrated that *P. americana* can act as a transport (paratenic) host for *Eimeria tenella*: cockroaches fed oocyst-contaminated material retained viable oocysts that subsequently infected chickens, providing a mechanistic pathway by which cockroaches may sustain coccidiosis cycles in poultry houses [73].

## 7. Cockroaches as Carriers of Human Pathogens in Community and Agricultural Settings

### 7.1. Poultry Farms and Salmonella

Poultry-focused epidemiological work indicates that cockroaches, together with other arthropods, participate in the environmental maintenance of *Salmonella* on farms [74]. Reviews of *Salmonella* in poultry highlight these pests as important vectors moving between litter, feed, carcasses, and structural refugia, thereby perpetuating environmental contamination even when bird-level infection appears controlled [75,76]. Experimental studies summarised by Shaji et al. have shown that cockroaches experimentally infected with *Salmonella* Typhimurium can contaminate table egg shells and contribute to within-flock spread, underscoring how environmental reservoirs and pest vectors complicate ‘farm-to-fork’ control under suboptimal biosecurity [75].

In a cross-sectional study from the Mekong Delta (Vietnam), Nguyen et al. sampled 3055 units from chickens, the farm environment and pest animals, including 287 cockroaches captured inside poultry houses and nearby dwellings [77], *Salmonella* was isolated from 31.3% of chicken cloacal swabs, 7.3% of environmental samples (e.g., litter, drinking water) and 2.4% (7/287) of cockroaches, with overlapping serovars detected across chickens, litter and pest animals, indicating shared transmission networks [77]. Antimicrobial susceptibility testing showed high levels of resistance among *Salmonella* isolates from all sources, with >50% resistant to tetracycline, ampicillin, and sulfamethoxazole/trimethoprim, and many strains exhibiting multi-class resistance [77].

On pig farms in Réunion Island, Tessier et al. conducted a cross-sectional survey of wild fauna and farm environments, isolating *Salmonella* from rodents, shrews, birds, and cockroaches around pig units [78]. Approximately one quarter of captured cockroaches carried *Salmonella*, and several serotypes were shared between cockroaches, pig faeces, and environmental samples, suggesting that peri-domestic insects contribute to the maintenance and redistribution of *Salmonella* in and around livestock housing [78]. Risk-factor analyses for persistent *Salmonella* contamination in broiler flocks similarly identify the presence of pests such as cockroaches and rodents, together with inadequate cleaning and disinfection, as significant predictors of environmental positivity, even after flock depopulation [79]. Controlled infection experiments have demonstrated prolonged *Salmonella* shedding by cockroaches, consistent with a capacity to contribute to faecal–oral transmission cycles on farms [75,80].

### 7.2. Ports and Border Crossings

Global trade and passenger shipping create opportunities for synanthropic cockroaches to be transported between countries on vessels and cargo, and reviews consistently identify ports and ships as high-risk but understudied sites for pathogen and AMR dissemination, with little structured cockroach monitoring [21,81].

One of the few detailed post-2016 investigations in a port environment comes from Indonesia, where Supryatno et al. conducted a descriptive survey of cockroaches on 24 cargo and passenger ships docking at Baubau Port [82]. The authors trapped 3196 cockroaches across ship galleys, bridges, decks, and bathrooms, with *P. americana* (69.5%) and *B. germanica* (29.6%) dominating the catch, and then tested a subset of 42 individuals for *Salmonella* spp. Culture and biochemical identification revealed that 95.3% (40/42) of examined cockroaches harboured *Salmonella* [82].

More recently, a xenon monitoring study from Shenzhen ports in southern China evaluated the feasibility of using imported flies and cockroaches for pathogen detection, providing rare contemporary data on cockroach-borne bacteria at border crossings [83]. Between October 2023 and April 2024, the authors analysed samples from imported flies and cockroaches, identifying *Musca domestica vicina* and *B. germanica* as the predominant species, accounting for 27.6% and 66.5% of captured specimens, respectively [83]. Among imported cockroaches, 6.5% were positive for *S. aureus*, and metagenomic sequencing revealed diverse bacterial communities, including several taxa associated with human disease, while highlighting broadly similar pathogen profiles across consignments from different Asian origins [83]. Although this study did not focus on AMR phenotypes, it demonstrates the feasibility of cockroach-based screening for bacteria at ports. Observations from Chinese port health surveillance suggest that intercepted cockroaches may already harbour diverse pathogens, including moulds, enteric bacteria, and hepatitis B virus, upon arrival. Thus, in their study of intestinal pathogens in food-related environments in Shanghai, Liu et al. noted that moulds, *E. coli*, *P. aeruginosa*, and hepatitis B virus (HBV) had been detected in both imported and locally captured cockroaches in the same metropolitan region, based on earlier port health surveillance reports [23]. These findings, together with systematic reviews summarising similar observations from other harbour settings, suggest that cockroaches arriving with cargo or on ships may already be contaminated with clinically significant pathogens before entering local urban ecosystems [8,21].

From a One Health standpoint, imported cockroaches constitute a plausible route for the international spread of *Salmonella*, *S. aureus*, and other pathogens, including potentially MDR lineages, but contemporary quantitative data on AMR profiles, resistance genes, and onward establishment in local cockroach or environmental populations are almost entirely lacking [8,83].

### 7.3. Households, Restaurants, and Warehouses

A recent narrative review concluded that food-borne pathogens, particularly *E. coli* O157:H7, *S. aureus*, and *Salmonella* spp., constitute approximately one-quarter of microbial taxa isolated from cockroaches globally [21]. Community surveys in Pakistan, Ethiopia, and Bangladesh provide quantitative data on bacterial carriage and resistance patterns in cockroaches from households, restaurants, and other food-related environments. [34,84,85]. In Lahore, Pakistan, Memona et al. collected 240 adult cockroaches (167 *P. americana*, 73 *B. germanica*) from houses and hospitals and screened both external surfaces and gut contents; eleven bacterial species were isolated, with *E. coli* most common on external surfaces (10.3%) and *P. aeruginosa* predominating in gut samples (19.9%) [86]. In Jimma town, Ethiopia, Solomon et al. sampled *B. germanica* from restaurant kitchens, cafeterias, and bakery environments, isolating a broad spectrum of enteric and opportunistic bacteria, including *E. coli*, *Klebsiella* spp., *Proteus* spp., *Salmonella* spp., and *S. aureus*, from both external washes and dissected gut contents [87]. Similarly, in Dhaka, Bangladesh, Naher et al. examined 450 *B. germanica* collected from hospitals, restaurants, and houses, isolating *Salmonella* spp., *Shigella* spp., *E. coli*, and *S. aureus* from both external and internal samples [88]. Across these sites, many isolates, particularly Gram-negative bacilli and *S. aureus*, showed frequent resistance to commonly used agents such as ampicillin, tetracycline, co-trimoxazole, and erythromycin, with formal multidrug resistance documented in Ethiopia and Bangladesh and strongly suggested in Pakistan [86,87,88].

In Shanghai, Liu et al. trapped 266 cockroaches from catering establishments, school kitchens, and other food-related venues, identifying 65.8% as *B. germanica* and 34.2% as *Periplaneta fuliginosa* using morphological and molecular tools [23]. Among 128 dissected digestive tracts, 28.1% (36/128) were positive for at least one intestinal pathogen, with sapovirus and norovirus as the predominant viruses, Shiga toxin-producing *E. coli* (STEC) the most frequently detected bacterium, and *Blastocystis hominis* the most common parasite [23]. Notably, nymphs had significantly higher pathogen detection rates than adults, and some cockroaches carried combinations of viruses, bacteria, and protozoa [23].

## 8. Mechanisms and Routes of Pathogen Transmission

### 8.1. Mechanical Transmission

Healthcare settings provide some of the clearest operational data on cockroach-mediated transfer of pathogens because these insects move repeatedly between wet environmental reservoirs (sinks, drains, waste bins) and high-touch clinical surfaces (bed rails, worktops, medication trolleys). Body-surface wash studies show substantial bacterial contamination of cockroach tarsi and ventral surfaces, but the absence of concurrent clinical and surface sampling limits inference about direct involvement in particular nosocomial outbreaks [10,15].

Mechanical transmission also includes aerosolization of faecal particles and exuviae, which act as carriers for cockroach-derived proteins, endotoxins, and potentially associated microbes. Cockroach allergens such as Bla g 1 and Bla g 2, found in saliva, faeces, and body fragments, are well-established drivers of allergic sensitisation and asthma, particularly in urban, low-income populations where cockroach infestation is common [89]. In a narrative review of indoor allergen exposures, Pham et al. and others (not specific to cockroaches) have highlighted that mechanical disturbance of infested areas can generate airborne particles in the respirable size range, allowing for inhalational exposure to allergen-laden dust [89]. Building on this concept, Kakumanu et al. combined laboratory and field measurements in low-income urban apartments in the United States, showing that female *B. germanica* excreted approximately 2900 EU of endotoxin per mg of faeces compared with 1400 EU/mg in males, and that cockroach-infested homes had significantly higher concentrations of Bla g 2 and endotoxin in settled dust and HVAC filters than uninfected homes [90]. After integrated pest management interventions that eliminated infestations, both endotoxin and allergen levels declined markedly, implying that ongoing cockroach activity continually resupplies airborne and dust reservoirs through mechanical shedding and faecal deposition [90]. Complementary metagenomic work by Ma et al. in five Chinese cities used 16S rRNA sequencing and microbial source-tracking to estimate that cockroach-associated bacteria contributed about 5.6% of the floor dust microbiome and 1.3% of the indoor airborne microbiome in infested homes, with potential pathogenic bacteria comprising up to 58.9% of cockroach microbiota. This study provides quantitative evidence that cockroach-derived bacteria are repeatedly dispersed into indoor air and dust, although it did not directly measure the viability of specific pathogens in airborne particles [91].

### 8.2. Biological Transmission

Experimental work integrating culture, microscopy, and molecular assays has characterised how *S. Typhimurium* colonises the *B. germanica* hindgut over time [92]. In Turner et al.’s study, adult German cockroaches were orally inoculated with *S. Typhimurium* and followed longitudinally; bacterial loads remained high in the hindgut over a week of observation, and microscopy revealed biofilm-like aggregates adherent to the gut lining rather than a uniform dispersion of cells, supporting the existence of stable micro-habitats for the pathogen [93]. A subsequent infection genomics analysis comparing multiple laboratory and field-derived *B. germanica* strains exposed to the same *S. Typhimurium* dose demonstrated that some field strains developed higher infection prevalence and shed more bacteria over time than long-term laboratory lines, suggesting that naturally evolved genetic variation in host pathways associated with immunity and gut physiology underpins differences in vector competence [94]. Together, these data indicate that the cockroach gut can function as a permissive and genetically variable habitat for *S. Typhimurium*, more akin to biological transmission than to passive mechanical carriage.

The interaction between *S. Typhimurium* and cockroach innate immunity is not simply a generic response to bacterial load, but exhibits pathogen and virulence factor specificity. Turner and Pietri fed adult *B. germanica* either live wild-type *S. Typhimurium*, live *E. coli*, heat-killed *S. Typhimurium*, or a live *S. Typhimurium* mutant lacking type III secretion systems, and quantified AMP gene expression in dissected guts 1 and 24 h post feeding (typically 4–5 cockroaches per treatment per time point) [95]. Robust induction of several AMP genes (including defensins, attacins, and blattellicin) occurred only after ingestion of live wild-type *S. Typhimurium*, whereas *E. coli*, heat-killed *S. Typhimurium*, and the type III secretion-deficient mutant elicited little or no AMP upregulation, despite similar exposure to bacterial pathogen-associated molecular patterns. These results imply that the cockroach gut immune system responds primarily to active colonisation processes involving type III secretion, rather than simply to high doses of luminal bacterial products, and raise the possibility that *S. Typhimurium* manipulates AMP responses in ways that favour its own persistence [95].

Microbiota-dependent structuring of the hindgut has emerged as a key determinant of *S. Typhimurium* persistence. In an elegant mechanistic study using germ-free and conventional *B. germanica*, Turner et al. discovered that the commensal gut microbiota induces discrete melanin plaques along the hindgut lumen; these plaques act as physical substrates on which *S. Typhimurium* forms dense aggregates, whereas *E. coli* shows minimal adhesion [96]. Deletion of the type 1 fimbrial adhesin gene *fimA* markedly reduced bacterial attachment to melanin deposits and diminished aggregate formation, and this *fimA* mutant was shed less efficiently in faeces than the wild-type strain, demonstrating that specific bacterial adhesins and microbiota-induced host structures cooperate to establish stable gut reservoirs [96]. Building on this structural insight, an iScience study modulated the cockroach gut microbiota with antibiotics and subsequent recolonisation, then quantified melanin deposition, *S. Typhimurium* loads, and AMP expression: disruption of the microbiota reduced melanin plaque formation, increased susceptibility to infection, and altered gut AMP transcription, whereas restoration of a diverse microbiota re-established melanin and partially restored colonisation resistance [96]. These experiments collectively support a model in which the commensal microbiota simultaneously creates physical niches (melanin plaques) and shapes the immune landscape that together determine whether *S. Typhimurium* is excluded or maintained as persistent hindgut aggregates.

High-resolution single-cell genomics and metatranscriptomics of the hindgut microbiota in the related omnivorous species *P. americana* revealed dense, spatially structured consortia of bacteria and archaea specialising in fermentation, hydrogen metabolism, and nitrogen cycling, underscoring that cockroach hindguts broadly are ecologically complex systems in which invading pathogens must integrate into or disrupt established metabolic networks [97].

These gut-level biological interactions have direct consequences for environmental contamination and for the efficacy of standard control measures. In a recent study, Van Hulzen and Pietri exposed German cockroaches to sublethal doses of an indoxacarb-based gel bait (Advion Evolution), then orally challenged surviving individuals with *S. Typhimurium* and monitored infection and shedding [98]. In one strain, prior sublethal bait exposure increased the prevalence of detectable infection 24 h after *S. Typhimurium* ingestion from 47% in unexposed controls to 77% in bait-exposed cockroaches, and infected survivors continued to excrete viable bacteria for several days [98]. These findings indicate that typical baiting regimens, which do not immediately remove all individuals from a population and may leave a subset of physiologically stressed but surviving cockroaches, can paradoxically increase susceptibility to infection and sustain faecal shedding, thereby prolonging the period during which cockroaches contaminate surfaces and food with enteric pathogens.

Biological interactions with *E. coli* illustrate that not all enteric bacteria behave equivalently in the cockroach gut and that developmental stage and microbiota strongly influence clearance. In a controlled infection model, Ray et al. orally infected *B. germanica* with several *E. coli* strains and measured bacterial titres over time; a laboratory K-12-derived strain was largely eliminated within 48 h, whereas a field isolate persisted in 80–100% of cockroaches for more than three days with little impact on host survival, indicating strain-dependent persistence [99]. Nymphs displayed greater capacity to clear certain *E. coli* strains than adults, despite similar expression of the antimicrobial effector lysozyme, suggesting that other immune pathways or microbiota-related mechanisms underlie developmental differences in susceptibility [99]. Crucially, gnotobiotic cockroaches reared with markedly reduced environmental microbiota showed impaired clearance of *E. coli* compared with conventionally colonised controls, directly implicating the native gut microbiota in colonisation resistance against exogenous enteric bacteria [99].

## 9. Knowledge Gaps and Future Research Directions

### 9.1. Strengthening Epidemiological Links

An ICU outbreak investigation involving MDR *E. cloacae* implicated persistent cockroach infestation as a potential driver of prolonged transmission over 20 months, while acknowledging concurrent environmental and patient-to-patient routes [100]. Future outbreak investigations will need to embed systematic cockroach sampling alongside patient, environmental, and wastewater surveillance to quantify attributable risk and to test whether targeted vector control meaningfully interrupts transmission chains [8,50].

Existing molecular epidemiological work illustrates the potential but also the current limitations of strain-level linkage between cockroach and human isolates. Similar clonal relatedness has been suggested between hospital cockroach isolates and clinical Enterobacterales in North Africa, but most studies rely on phenotypic resistance profiles and limited genotyping rather than whole-genome sequencing (WGS) [40,44]. Dedicated WGS-based analyses of cockroach isolates, such as complete genomes of nosocomial strains recovered from *B. germanica* in a Bangladeshi hospital, have shown that insects can carry multiple plasmid-encoded resistance genes, but these datasets are rarely integrated with contemporaneous patient isolates [46].

Because most cockroach studies are cross-sectional snapshots, longitudinal sampling integrated with human and environmental data, similar to the time-series designs already used in poultry production to track pest-related *Salmonella* persistence, is needed to clarify acquisition, persistence, and the impact of control measures on pathogen and AMR dynamics [75,101].

### 9.2. Understanding AMR Ecology in Cockroaches

Key ecological processes governing antimicrobial resistance in cockroaches, such as the rates at which they acquire novel resistance determinants from food, sewage, animal waste, or hospital surfaces, the conditions under which ARGs and resistant strains persist or are lost from the gut, and the frequency of plasmid transfer among co-resident microbes, remain largely unquantified [24,33]. New experimental evolution and mesocosm designs that expose cockroaches to realistic gradients of antibiotics, disinfectants, and organic waste, mirroring hospital sewer systems, informal settlements, and intensive poultry operations, are needed to model ARG acquisition, maintenance, and loss across cockroach life stages and generations [37,50]. Another major gap is the poor integration of cockroach resistome data with parallel information from humans, livestock, and the environment; farm and market studies in Africa and Asia rarely include cockroaches, and even when they do, harmonised comparative genomic analyses of ARG and plasmid repertoires across reservoirs remain exceptional [102,103,104,105].

Interactions between insecticide resistance, heavy metal exposure, and antibiotic resistance in cockroaches are another underexplored dimension of AMR ecology. While phenotypic resistance to pyrethroids and other insecticides is widely reported in *B. germanica* and *P. americana*, hardly any studies have characterised insecticide resistance mechanisms, co-residing ARGs, and metal- or biocide-resistant genes in cockroach microbiomes or mobile genetic elements [8,37]. Environmental microbiology research from intensive livestock systems shows that metal and antibiotic resistance genes often co-localise on the same plasmids, suggesting that non-antibiotic stressors may co-select for MDR phenotypes [102,106]. Comparable analyses targeting cockroach-associated bacteria could reveal whether insecticide and metal exposures in urban sewers, hospital basements, and farm structures promote co-selection of ARGs, with important implications for both pest control and AMR mitigation policies [4].

### 9.3. Optimisation of Surveillance Tools

A key operational challenge is the lack of standardised entomological and microbiological protocols for cockroach-based surveillance of pathogens and AMR. Reviews of hospital and community studies document wide variation in species identification methods, trapping strategies (sticky traps, manual capture, baited traps), sampling units (individual versus pooled insects), and anatomical targets (whole-body homogenates, cuticle swabs, gut contents), all of which can markedly influence estimates of prevalence and bacterial load [7,10]. Microbiological methods are equally heterogeneous, ranging from simple culture on non-selective media to selective enrichment and ESBL screening plates, and from limited disc diffusion testing to comprehensive EUCAST/CLSI-based panels, thereby hampering between-study comparisons and meta-analysis of AMR patterns [40,50]. This heterogeneity has direct implications for the comparability of resistance data: studies using selective ESBL/carbapenemase screening media or targeted PCR will often report higher proportions of resistant isolates than studies relying on non-selective culture, and different antibiotic panels and breakpoint systems (CLSI vs. EUCAST) can change MDR classification. In this review, we therefore treat MDR/ESBL/carbapenemase values as study-specific indicators and avoid inferring quantitative differences between countries or settings solely from the reported percentages in Table 1 and Table 2. Standard operating procedures tailored to cockroach surveillance are therefore urgently needed, ideally endorsed by infection prevention and One Health authorities, to ensure that data are comparable across sites and time.

Recent advances in rapid microbial identification and genomics, particularly MALDI-TOF mass spectrometry and WGS, offer promising tools for cockroach-based surveillance but require better integration into scalable workflows. MALDI-TOF MS can provide high-throughput species-level identification of cockroach-derived isolates, serving as an efficient entry point for subsequent resistance testing and genomic characterisation, while WGS, including relatively low-cost nanopore platforms, can reveal detailed resistome and mobile genetic elements that are invisible to conventional phenotyping. However, only a minority of cockroach surveys currently apply even basic PCR screening for key ESBL and carbapenemase genes [7,39]. Future work should pilot tiered surveillance pipelines that couple inexpensive culture-plus-PCR screening of pooled cockroach samples with targeted WGS of priority isolates to maximise information yield under resource constraints [37,50].

Cost-effective strategies for low- and middle-income settings, where cockroach infestations and AMR burdens are often highest, remain largely undeveloped. An Indonesian ESBL surveillance study of *P. americana* demonstrated that modestly sized but well-structured samples are sufficient to detect site-specific differences in ESBL frequency and genotype profiles [51]. Yet most AMR surveillance programmes in such settings still ignore insects, focusing instead on clinical isolates and occasionally wastewater, despite calls to include synanthropic pests as low-cost sentinels [4,50].

### 9.4. Balancing Vector Control and Drug Discovery

An emerging but delicate frontier concerns the dual identity of cockroaches as both public health pests and potential sources of novel antimicrobial compounds [107]. Complementary studies have shown that bacteria isolated from cockroach guts and cuticles can produce secondary metabolites with antagonistic activity against food-borne and environmental pathogens, suggesting that these microbiomes constitute reservoirs of potentially exploitable bioactive molecules [9,108]. Microbiome-informed screening has yielded cockroach-derived peptide *Candidates* with in silico activity profiles against MDR pathogens, suitable for synthetic production and experimental evaluation [27].

However, the growing interest in cockroaches as a source of new antimicrobials risks creating conceptual and practical tension with the imperative to control these insects as vectors and reservoirs of AMR. Reviews of insect-derived AMPs emphasise their promise as alternatives or adjuncts to conventional antibiotics, but most studies still rely on broad screening of insect tissues or symbionts without clear guidance on how to decouple drug discovery from ongoing environmental infestation [109,110,111]. In cockroaches specifically, bioprospecting has often used field-collected insects from highly contaminated environments, raising biosafety concerns and potentially undermining infection control messaging if the public or facility managers perceive cockroaches as “useful” organisms to be preserved [9]. Future research should prioritise ex situ approaches, such as maintaining pathogen-free laboratory colonies, cryopreserved cell lines, or culturable symbiont libraries, and genome-based mining of existing cockroach microbiome datasets, thereby reducing dependence on persistent infestations in hospitals, farms, and households [27,112].

Operationally, integrating vector control and drug discovery agendas requires clear governance frameworks that prevent trade-offs detrimental to infection prevention and control (IPC). Cockroach-focused integrated pest management (IPM), combining environmental sanitation, structural exclusion, targeted baiting, and insecticide resistance management, should remain the primary objective in clinical and food production settings, with bioprospecting activities constrained to research facilities or carefully regulated field collections that do not interfere with IPM implementation [8,50].

## 10. Conclusions

Synanthropic cockroaches, particularly *B. germanica* and *P. americana*, clearly emerge from current evidence as more than nuisance pests: they are persistent, mobile reservoirs in which complex microbiota, high-risk bacterial, fungal, viral, and parasitic pathogens and diverse antimicrobial-resistant determinants converge across hospitals, households, food premises, farms, and ports. Culture-based and molecular data show frequent carriage of MDR and, in some cases, XDR Enterobacterales, non-fermenters, and Gram-positive cocci, including ESBL-, carbapenemase- and *mcr*-bearing lineages that mirror local clinical and livestock resistome. Experimental infection models further demonstrate that key enteric pathogens such as *Salmonella* can colonise the cockroach hindgut, form stable aggregates, and be shed over prolonged periods, blurring the traditional boundary between purely mechanical transmission and biological reservoir status. Parallel work on fungal and parasitic contamination, including azole-resistant *Candida* and soil-transmitted helminths, extends this risk profile beyond bacteria and supports a One Health view in which cockroaches link human, animal, and environmental pathogen pools along food chains and transport networks. Collectively, these data justify treating cockroaches as epidemiologically relevant vectors and sentinels within infection prevention and control, food safety, and biosecurity frameworks.

At the same time, major gaps constrain the precise quantification of their contribution to infection and AMR dynamics. Strain-level genomic linkage between cockroach and clinical or livestock isolates remains sporadic, longitudinal data are scarce, and standardised trapping, sampling, and microbiological protocols are lacking, limiting comparability between studies and the operational use of cockroach-derived data in AMR surveillance. Future work should integrate systematic cockroach sampling into hospital, farm, and port surveillance systems, pair culture-based screening with targeted genomic characterisation of priority isolates, and explicitly examine interactions between microbiota, insecticide resistance, environmental co-selectors, and ARG exchange in cockroach-associated communities. Efforts to exploit cockroach microbiomes and immune effectors as sources of novel antimicrobial compounds must be clearly separated from, and never compromise, rigorous integrated pest management in clinical and food production settings. Addressing these research and governance priorities will enable cockroach control and surveillance to be more effectively embedded within One Health AMR strategies, helping to reduce environmental dissemination of high-risk pathogens while safely harnessing any potential translational benefits of cockroach–microbiota biology.

## Data Availability

No new data were created or analyzed in this study. Data sharing is not applicable to this article.

## Abbreviations

The following abbreviations are used in this manuscript:

Abbreviation	Definition
16S rRNA	16S ribosomal RNA gene
3GC	Third-generation cephalosporin
AMP	Antimicrobial peptide
AMR	Antimicrobial resistance
ARG	Antimicrobial resistance gene
Bla g 1	*Blattella germanica* allergen 1
Bla g 2	*Blattella germanica* allergen 2
CLSI	Clinical and Laboratory Standards Institute
COVID-19	Coronavirus disease 2019
CRe	Cephalosporin-resistant Enterobacterales
ESBL	Extended-spectrum β-lactamase
EU	Endotoxin unit
EUCAST	European Committee on Antimicrobial Susceptibility Testing
HBV	Hepatitis B virus
HVAC	Heating, ventilation, and air conditioning
ICU	Intensive care unit
IMD	Immune deficiency pathway
IPC	Infection prevention and control
IPM	Integrated pest management
MALDI-TOF	Matrix-assisted laser desorption/ionisation time-of-flight
MBL/MβL	Metallo-β-lactamase
MDR	Multidrug-resistant
MRSA	Methicillin-resistant *Staphylococcus aureus*
MS	Mass spectrometry
NDM	New Delhi metallo-β-lactamase
NICU	Neonatal intensive care unit
PCR	Polymerase chain reaction
RT-PCR	Reverse-transcription polymerase chain reaction
RT-qPCR	Reverse-transcription quantitative PCR
SARS-CoV-2	Severe acute respiratory syndrome coronavirus 2
STEC	Shiga toxin–producing *Escherichia coli*
WGS	Whole-genome sequencing
WHO	World Health Organization
XDR	Extensively drug-resistant
